# Enhancing Antimicrobial and Antioxidant Properties of Chitosan-Based Films with 1-Methylimidazolium-Chitosan

**DOI:** 10.3390/polym17192608

**Published:** 2025-09-26

**Authors:** Carolina Muñoz-Nuñez, Yoleida Quiroz-Pereira, Alexandra Muñoz-Bonilla, Marta Fernández-García

**Affiliations:** 1 Instituto de Ciencia y Tecnología de Polímeros (ICTP-CSIC), C/Juan de la Cierva 3, 28006 Madrid, Spain; 2 Facultad de Ciencias Químicas, Universidad Complutense de Madrid, Avenida Complutense s/n, Ciudad Universitaria, 28040 Madrid, Spain; 3 Interdisciplinary Platform for Sustainable Plastics Towards a Circular Economy-Spanish National Research Council (SusPlast-CSIC), 28006 Madrid, Spain

**Keywords:** chitosan, imidazole, chitin nanowhiskers, chemical modification, antimicrobial, antioxidant, films

## Abstract

The design and the synthesis of functional films with enhanced functionality represent a significant step forward in sustainable material development due to their potential applications. In this study, a novel chitosan derivative (CS-MeIm) was synthetized by chemically modifying chitosan (CS) structure with 1-methyl-1H-imidazole (MeIm), a heterocyclic compound known for its biological properties. This functionalization not only enhances the intrinsic capabilities of CS but also provides a strategic platform for advanced material engineering. The modified compound, CS-MeIm, was incorporated at 10 wt% into films based on CS matrix, which was also reinforced with 1 or 5 wt% of chitin nanowhiskers (ChNw), to improve their functionality for its potential applications. The fabrication process was optimized to ensure the homogeneity and the structural integrity of the films, which were extensively evaluated to study their thermal stability, mechanical integrity, and bioactivity. The incorporation of the imidazole ring into the CS backbone provided a marked enhancement in antioxidant capacity from 3 to 15 μmol Trolox/gram of film; and excellent antimicrobial activity against common microbes, particularly against E. coli with an efficacy of 99.999%. The findings reveal that this chemical modification not only raises the intrinsic properties of CS but also introduces a versatile platform for creating biodegradable films with high functionality.

## 1. Introduction

The proliferation of pathogenic microorganisms presents a significant challenge in sectors such as public health [1] and the food industry [2]. The increasing resistance to antibiotics underscores the urgent need for innovative, effective, and sustainable antimicrobial strategies [3,4]. In this context, materials with advanced functional properties emerge as promising solutions to fight microbes while ensuring environmental safety.

Heterocyclic compounds, such as pyridines [5], pyrroles [6], or imidazoles [7], have demonstrated great potential due to their unique structures and bioactive characteristics [8,9]. Specifically, the imidazole molecule, whose ring contains two nitrogen atoms and an aromatic system, exhibits antioxidant and antimicrobial properties that make it a valuable component in some formulations [10]. The imidazole ring can contribute to antioxidant activity through electron donation, thereby preventing oxidative damage, a crucial advantage in oxidation-sensitive applications like food packaging materials [11]. In addition, its structure enables electrostatic interactions and bonding with biomolecules, which is relevant for enhancing biocompatibility and functionality. When one of the nitrogen atoms in the imidazole ring is quaternized [12], the resulting positive charge could reinforce the electrostatic interactions and produces a synergistic effect that enhances antioxidant activity. In terms of antioxidant activity, the quaternized system can interact with free radicals through electronic resonance and the positive charge of the imidazolium cation, stabilizing radical species and contributing to the integrity of materials exposed to oxidative conditions [13]. Simultaneously, this positive charge enhances its antimicrobial properties by facilitating interactions with bacterial membranes, which have negatively charged surfaces. This electrostatic interaction disrupts the microbial membrane, allowing the entry of active compounds and ultimately inhibiting the growth of pathogenic bacteria [14].

The use of these molecules for polymer functionalization offers an effective way to enhance the biological properties of these materials [15,16,17,18]. The unique properties of imidazole and its derivatives could allow the creation of polymer systems with specific functions designed to meet the requirements of food active packaging applications, aiming to extend the shelf life of food products. Indeed, the demand for sustainable and active materials is growing, and this approach provides a pathway to address critical challenges related to food preservation and safety. In this context, Chitosan (CS) and derivatives have emerged as very promising biopolymers in food packaging area. CS, a biopolymer derived from the deacetylation chitin, is renowned for its biocompatibility [19] and biodegradability [20], and it is found in the exoskeletons of some crustaceans, crabs, or insects from natural sources, making it a sustainable and cost-effective option [21]. One of the most important characteristics of CS is its film-forming ability [22], enabling the formation of continuous and robust films that are ideal for packaging applications [23]. Additionally, its structure contains amino groups that can be easily chemically modified, allowing for versatile and adaptable functionalization [24,25], improving its bioactive properties, including enhanced oxidation resistance and antimicrobial capacity [18], two critical aspects for prolonging the shelf life of food products. Mechanical and barrier properties are also key aspects in food packaging; films and some nano-scale reinforcements have emerged as valuable components in polymer matrices. Among these, chitin nanowhiskers (ChNw) produced by the acid hydrolysis of chitin powder represent an interesting option due to their dimensions such as needle-like morphology [26], large surface area [27], and strong hydrogen bonding capacity [28], which enables them to form robust interactions with CS matrix. The addition of ChNw enhances the mechanical stability and flexibility of the composite but also improves its barrier properties against oxygen and moisture [29], factors crucial for applications in packaging and preservation.

In this study, chitosan (CS) has been modified with 1-methyl-1H-imidazole (MeIm) to create a functional derivative to be used as an additive in the preparation of CS-based films, introducing some biological properties. Moreover, the influence of reinforcing ChNw on the structure and performance of the films will be evaluated, enhancing their stability and mechanical strength.

This combination of additives and reinforcements would provide a robust platform for developing innovative materials solutions to address the challenges of food safety and environmental sustainability, particularly in the field of active and biodegradable food packaging.

## 2. Materials and Methods

### 2.1. Materials

Chitosan from shrimp shells (deacetylation degree > 75%), chitin form shrimp shells (Ch), 1-methyl-1H-imidazole (MeIm, 99%), 2-bromoacetic acid (97%), glycerol (Gly, 99%), ethyl-3-(3-dimethylaminopropyl) carbodiimide (EDC, ≥98%), *N*-hydroxysuccinimide (NHS, 98%), 2-(*N*-morpholino) ethane sulfonic acid (MES solution), sodium chloride (NaCl), and 2,2-diphenyl-1-picrylhydrazyl (DPPH) were purchased from Merck (Darmstadt, Germany). All the organic solvents were of analytical reagent grade, acetonitrile (CH_3_CN), hydrochloric acid (HCl, 37%), tetrahydrofuran (THF), and methanol (MeOH) were provided by Scharlau (Barcelona, Spain). Deuterated dimethyl sulfoxide (DMSO-d6) was acquired from Merck. Cellulose dialysis membranes (CelluSep T1) were purchased from Membrane Filtration Products, Inc. (Seguin, TX, USA). Chitin nanowhiskers (ChNw) were synthesized according to our previous work [30].

For the antibacterial evaluation, saline solution (NaCl, suitable for cell culture, BioXtra, (Seneffe, Belgium) and phosphate-buffered saline (PBS) powder (pH 7.4) were sourced from Merck. BBL Mueller–Hinton broth microbial growth medium was obtained from Becton, Dickinson, and Company (Heidelberg, Germany), while the 96-well microplates and Columbia agar plates enriched with 5% sheep blood were acquired from Thermo Fisher Scientific (Dreieich, Germany). The following bacterial strains were utilized: *Escherichia coli* (*E. coli*, ATCC 25922), *Listeria innocua* (*L. innocua*, ATCC 33090), *Staphylococcus epidermidis* (*S. epidermidis*, ATCC 12228), *Staphylococcus aureus* (*S. aureus*, ATCC 29213), *Methicillin-resistant Staphylococcus aureus* (*MRSA*, ATCC 43300), *Enterococcus faecalis* (*E. faecalis* ATCC 29212), *Pseudomonas aeruginosa* (*P. aeruginosa*, ATCC 27853), and *Candida albicans* (*C. albicans*, ATCC 200955), all obtained from Oxoid^TM^ (Thermo Fisher Scientific, Basingstoke, UK).

### 2.2. Synthesis of 3-(Carboxymethyl)-1-methyl-1H-imidazol-3-ium Bromide (MeImB)

**Scheme 1:**
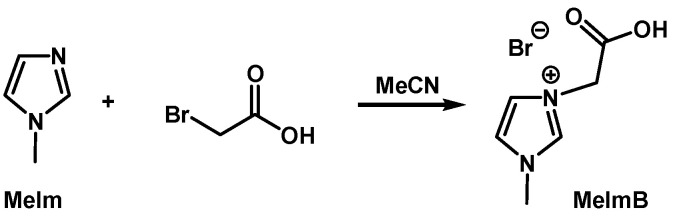
Synthesis of 3-(carboxymethyl)-1-methyl-1H-imidazol-3-ium bromide (MeImB).

First, 50 mmol of MeIm were dissolved in 25 mL of acetonitrile. Once dissolved, 50 mmol of bromoacetic acid were added to obtain the quaternized ammonium with a bromide ion. The reaction was stirred under an inert argon atmosphere and maintained at reflux for 24 h [31]. After this period, the solvent was removed using a rotary evaporator. The white solid was redissolved in the minimum possible amount of methanol, precipitated into an excess of cold tetrahydrofuran, and recovered by filtration. The MeImB product (see Scheme 1) was characterized by ^1^H-NMR and ^13^C-NMR.

### 2.3. Synthesis of Chitosan Derivative (CS-MeImB)

**Scheme 2:**
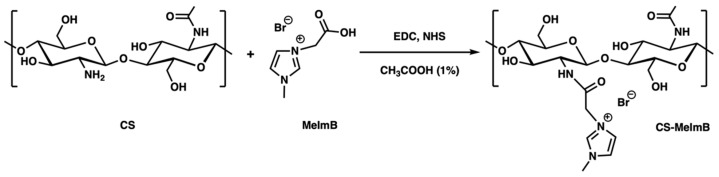
Synthesis of chitosan derivative (CS-MeImB).

Using the recently synthesized compound, MeImB was incorporated into CS through N-acylation, employing the EDC/NHS coupling system (Scheme 2). First, 0.5 g (1 meq of amine groups) of CS were dissolved in a 1% (*v*/*v*) aqueous glacial acetic acid solution. Simultaneously, 1.35 g of MeImB (2 meq) were dissolved in 15 mL of MES buffer solution (0.1 M) along with 1.18 g of EDC (2 meq) and stirred for 20 min [32]. After this period, 0.7 g of NHS (2 meq) were added, and stirring continued for 10 additional min. After this time, the MeImB solution was dropwise added to the CS solution, and the reaction was stirred for 24 h at room temperature [33]. After the corresponding dialysis process against water for 3 days (MW_CO_ of 12 kDa) and subsequent lyophilization, CS-MeImB was obtained and characterized with several techniques.

### 2.4. Synthesis and Characterization of Chitin Nanowhiskers (ChNw)

Chitin powder was acidified via hydrolysis in aqueous media to obtain ChNw. Briefly, 10 g of Ch were suspended in 500 mL of 3 M hydrochloric acid at 100 °C for 90 min under reflux conditions with continuous mechanical agitation. The acid suspension was then diluted in deionized water and subjected to three sequential centrifugation cycles of 10 min (12,000 rpm) to eliminate residual acid. The purified nanowhiskers were dialyzed (6–8 kDa MW_CO_ membrane) against distilled water for 4 days to neutralize pH, followed by freeze-drying to yield the final amount of ChNw [30]. The morphology of ChNw was analyzed using Field Emission Scanning Electron Microscopy (FE-SEM) on a Hitachi SU 8000 (Hitachi High-Technologies, Tokyo, Japan) in transmitted electron imaging mode. ChNw was dispersed in distilled water, and a drop was deposited onto a carbon-coated copper grid and allowed to dry at room temperature. The dried sample was subsequently coated with gold/palladium (80/20) before visualization. The longitudinal dimension of the nanowhiskers was measured using ImageJ 1.53K.

### 2.5. Film Preparation

Once the CS derivative was synthesized, it was used as an additive in the preparation of CS-based films, reinforced with previously obtained ChNws [30]. The films were produced with a base of 85% (*w*/*w*) polymer and 15% (*w*/*w*) glycerol as a plasticizer [34]. The polymer part was dissolved in a 1% (*v*/*v*) glacial acetic acid solution to obtain a final concentration of 1% (*w*/*v*). This solution included CS as the base matrix, the CS-MeImB additive, and varying amounts of ChNws to reinforce the film. All component quantities are detailed in Table 1. Glycerol was added to the solution, and the mixture was stirred for 24 h at room temperature. After this time, 50 mL of each solution were transferred to a Petri dish and allowed to dry by casting, resulting in the required films.

### 2.6. Characterization

#### 2.6.1. Nuclear Magnetic Resonance (NMR) Spectroscopy

NMR spectra (^1^H and ^13^C) were recorded at room temperature using a Bruker Avance III HD-400AVIII spectrometer (Bruker, Rheinstetten, Germany). The MeImB molecule was analyzed using DMSO-d6 as the solvent, while a 1% CD_3_COOD solution in D_2_O was used for the analysis of both polymers, CS and CS-MeImB.

#### 2.6.2. Infrared Spectroscopy

The infrared spectra analysis was performed using a Perkin Elmer Spectrum Two instrument (PerkinElmer, Waltham, MA, USA) equipped with an attenuated total reflection module (ATR-FTIR). A background spectrum was collected before each sample measurement. Infrared spectra were recorded over a spectral range of 450 to 4000 cm^−1^ with a resolution of 4 cm^−1^.

#### 2.6.3. Elemental Analysis

A LECO CHNS-932 model PNT01 analyzer (LECO Corporation, St. Joseph, MI, USA) was utilized to perform the elemental analysis, which quantified the percentages of carbon, nitrogen, sulfur, and hydrogen in the samples. The acquired data, the mass ratios between carbon and nitrogen (C/N), were then used to determine the degrees of acetylation (DA) in chitosan and the degree of substitution (DS) in the chitosan modified with MeImB (CS-MeImB) (Equations (1) and (2)) [35].

(1) CN=100×n1×Mc+DA×n2×Mc100×n3×MN

(2) CN=100×n1×Mc+DA×n2×Mc+(DS×n4×Mc)100×n3×MN+(DS×n5×MN)

where M_C_ and M_N_ are the molar masses of carbon and nitrogen, respectively; n_1_ = 6, n_2_ = 2 and n_4_ = 4 are the number of carbons of deacetylated CS, acetamido group, and imidazole incorporated to Cs, respectively; and n_3_ = 1 and n_5_ = 2 are the number of nitrogen of CS and imidazole group, respectively.

#### 2.6.4. Thermogravimetric Analysis

Thermogravimetric analysis was performed using a TGA Q500 analyzer (TA Instruments, New Castle, DE, USA). The thermograms were obtained from room temperature to 800 °C at a heating rate of 10 °C/min in an air atmosphere (50 cm^3^/min). Calibration of the instrument for both temperature and weight was carried out following standard procedures.

#### 2.6.5. Differential Scanning Calorimetry

The thermal behavior of the CS and its derivative, CS-MeImB, was studied with a TA Instruments (New Castle, DE, USA) DSC Q2000 differential scanning calorimeter, equipped with a refrigerated cooling system under N_2_ atmosphere. The measurements were equilibrated at 0 °C and heated to 400 °C at 10 °C/min. The temperature scale was calibrated from the melting point of high-purity chemicals (lauric and stearic acids, and indium). Samples were prepared in sealed aluminum pans and weighed in an electronic balance.

#### 2.6.6. Zeta Potential Measurements

The zeta potential of the CS and CS-MeImB was measured at physiological pH and 25 °C using a Zetasizer Nano series ZS (Malvern Instruments Ltd., Worcestershire, UK). For the preparation of the samples, the polymers were dissolved in the aqueous medium at a concentration of 1 mg/mL. The mixture was vortexed and sonicated to ensure homogeneity prior to analysis. The Smoluchowski equation was used to calculate electrophoretic mobility. The results are reported as the average from ten separate measurements.

#### 2.6.7. X-Ray Diffraction

X-ray diffraction measurements were performed on CS and CS-MeImB at room temperature in reflection mode using a Bruker D8 Advance diffractometer equipped with a PSD Vantec detector (Bruker, Madison, WI, USA). CuKα radiation (λ = 0.15418 nm) was employed, operating at a voltage of 40 kV. The samples were locked on an appropriate holder and scanned over a range from 5° to 55° (2θ) with a step size of 0.02° and a collection time of 10 s per step.

#### 2.6.8. Yellowness Index

The yellowness index (YI) of the films was evaluated following the ASTM D1925-70 standard [36]. The transmittance measurements were carried out using a PerkinElmer LAMBDA 1050+ UV/Vis/NIR spectrophotometer (PerkinElmer, Waltham, MA, USA), and the YI was calculated using the following equation:

(3) $$YI=\frac{100\times \left(C_{x}X-C_{z}Z\right)}{Y}$$

where X, Y, and Z represent the CIE tristimulus values, while C_x_ (1.3013) and C_z_ (1.1498) are coefficients determined by the illuminant (D65) and the observation angle (10°).

#### 2.6.9. Water Vapor Transmission

The water vapor transmission (WVT) of the F-CS and F-CS-MeImB films was evaluated to determine their potential as moisture barriers, which is crucial for reducing microbial growth. Additionally, the influence of incorporating ChNw at concentrations of 1% and 5% was assessed.

WVT assay was conducted using ASTM E96-00e1 [37], the films studied were sealed over cups with a 10 mm diameter opening, containing 2 g of dry silica as a desiccant (relative humidity, RH 0%). These cups were then placed inside a desiccator maintained at 22 °C, containing a saturated sodium sulfate solution to establish a RH of 70%. The initial weight of each cup was recorded for 24 h, followed by periodic weight measurements to recorded changes in the moisture [38,39]. Three replicates of each film were analyzed to ensure statistical reliability. The weight against of time, and the WVT was determined from the slope of the linear regression, normalized by the exposed area and film thickness, using Equation (3). This normalization ensured an accurate comparison of permeability values across different film samples.

(4) $$WVT=\left(\frac{slope(g/h)}{A\left(m^{2}\right)\times t(h)}\right)\times thickness\left(m\right)$$

#### 2.6.10. Mechanical Properties

Tensile testing of the CS and CS-MeImB films was conducted utilizing a DX2000 QTest Elite MTS dynamometer (MTS Systems, Eden Prairie, MN, USA) at room temperature to evaluate their mechanical properties. Operating at a stretching rate of 10 mm/min and with a 100 N load cell. Dog-bone specimens with a gauge length of 20 mm and a width of 2 mm were used for the measurements. Stress–strain measurements were used to calculate the ultimate tensile strength, Young’s modulus, and elongation at break values. Each film was tested with a minimum of five specimens.

#### 2.6.11. Antioxidant Properties

The antioxidant activity of the CS and CS-MeImB was evaluated using the DPPH (2,2-diphenyl-1-picrylhydrazyl) method. A concentration of 1 mg of each sample was placed in 1 mL of DPPH solution. The DPPH solution was prepared by dissolving 6 mg of DPPH in 250 mL of methanol, stored at 4 °C, and protected from light to maintain stability. The absorbance of the DPPH solution was measured at 517 nm using a BioTek^®^ SYNERGY HTX multi-mode reader spectrophotometer (Agilent Technologies, Winooski, VT, USA) before and after the addition of the samples, at different times. The decrease in absorbance was used to calculate the percentage of DPPH scavenging activity, inhibition percentage, according to the following Equation (4):

(5) $$I\left(\%\right)=\left(\frac{A_{control}-A_{sample}}{A_{control}}\right)\times 100$$

where *A_control_* is the absorbance of the control (DPPH solution without samples), and *A_sample_* is the absorbance of the DPPH solution after adding the samples.

In the same way, the antioxidant capacity of both CS and CS-MeImB films was studied using the same DPPH method. 1 mL of DPPH solution was added to a sample of 10 mg of each film. The resulting mixture was incubated for a specific period and was measured at different times at 517 nm to see the interaction between the DPPH radicals and the films.

To standardize the results, radical scavenging activities of the samples were expressed as equivalents of Trolox. A calibration curve was generated by plotting Trolox concentrations against their corresponding inhibition percentages (I%), allowing for the quantification of the antioxidant capacity of the tested materials.

#### 2.6.12. Antimicrobial Properties

The antimicrobial properties of CS and CS-MeImB were assessed using the Minimum Inhibitory Concentration (MIC), following a standard broth dilution method according to the Clinical Laboratory Standards Institute (CLSI) [40]. The following microbial strains were used in the study: *S. aureus*, *S. epidermidis*, MRSA, *L. innocua*, *E. faecalis*, *E. coli*, *P. aeruginosa* and *C. albicans*. Bacterial and fungal strains were grown on 5% sheep blood Columbia agar plates at 37 °C for 24 h and 48 h, respectively. 

Initially, microbial suspensions were freshly generated in saline solution to obtain a final concentration of 10^8^ colony-forming units (CFU) per mL, corresponding to a turbidity near 0.5 on the McFarland scale. These initial suspensions were diluted to a concentration of ca. 10^6^ CFU/mL using fresh Mueller-Hinton broth. For this purpose, 2 mg of each sample (CS and CS-MeImB) were dissolved in 1 mL of Mueller–Hinton broth. In a 96-well round-bottom microplate, 100 µL of this polymer solution was added into the first well, followed by the broth addition of 50 µL to the other wells. Serial dilutions were performed across the plate at a ratio of 1:2. Following this, 50 µL of the microbial suspension was introduced into each well, leading to a total volume of 100 µL and a final microbial concentration of ca. 5 × 10^5^ CFU/mL.

The microplates were then incubated at 37 °C for 24 h and 48 h for bacterial and fungal strains. After incubation, the MIC was determined by visually inspecting the wells for bacterial growth. The lowest concentration of the sample that exhibited no visible growth was recorded as the MIC for each bacterial strain. All evaluations were conducted in at least three replicates.

Subsequently, the antimicrobial properties of the CS and CS-MeImB films were assessed using a similar methodology adapted from the E2149-20 standard method of the American Society for Testing and Materials (ASTM) [41] against the same microbial strains. 10 mg of each film were placed into 9 mL of PBS. Following this, 1 mL of a microbial suspension of ca. 10^6^ CFU/mL was added to the film solution. The tubes were shaken at 100 rpm for 24 h. After incubation, the resulting solutions were grown on 5% sheep blood Columbia agar plates at 37 °C for 24 h and 48 h for bacterial and fungal strains, respectively. The reduction percentage was determined by comparison with the CFU of the control by the plate counting methodology. The measurements were made at least in triplicate.

### 2.7. Statistical Analysis

All experiments were conducted in triplicate to ensure consistency. A one-way analysis of variance (ANOVA) was performed to evaluate significant differences in properties among the various groups, followed by post hoc Tukey’s test, with a significance level set at *p* ≤ 0.05.

## 3. Results and Discussion

Initially, the molecular weight of the CS was measured by viscometry according to the literature [42], the intrinsic viscosity of CS was measured in 0.1 M acetic acid with 0.2 M NaCl at different concentrations. The Mark–Houwink equation [η] = KMw^a^ was used to calculate the molecular weight. The value of K and a of 1.81 × 10^−3^ cm^3^/g and 0.93, respectively, were used [43]. The intrinsic viscosity was determined as 264 ± 7 cm^3^/g; therefore, the Mw is the range of 357 ± 10 kDa. Then, this CS was modified with MeImB. The successful incorporation of MeImB was confirmed through spectroscopic techniques as will be shown below. This modification not only altered the chemical structure of the polymer but also resulted in a significant improvement in its physicochemical properties. The resulting polymer presents excellent solubility in aqueous media, achieving the primary objective of the modification. Specifically, the zeta potential of the modified compound was determined to be 49.2 ± 4.7 mV, indicating a highly positive surface charge.

### 3.1. Synthesis and Characterization of Chitosan Derivative

**Figure 1:**
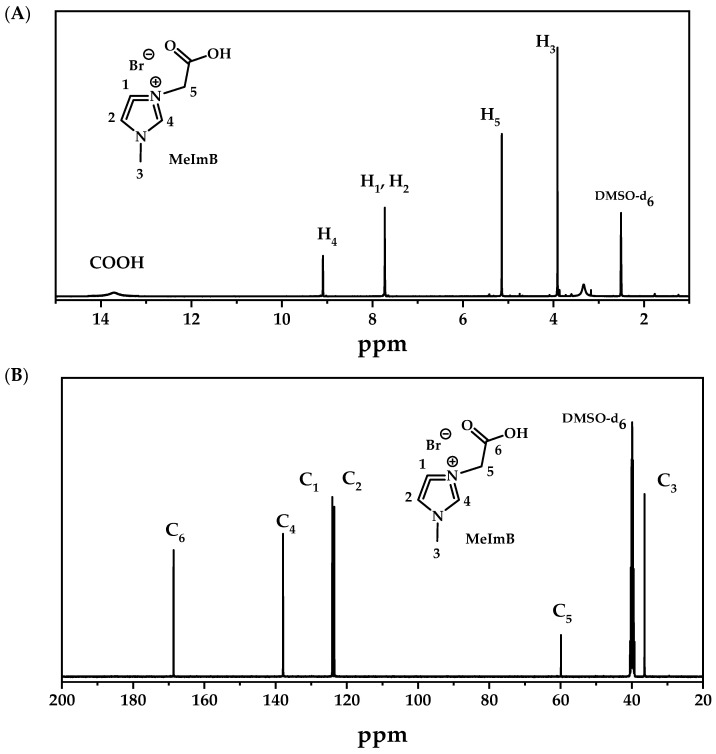
(**A**) ^1^H NMR and (**B**) ^13^C NMR spectra of MeImB.

Firstly, the compound 3-(carboxymethyl)-1-methyl-1H-imidazol-3-ium was successfully synthesized, and its chemical structure was confirmed by ^1^H NMR and ^13^C NMR spectroscopies (see Figure 1). For the MeImB molecule, the ^1^H NMR signals are observed at: δ (ppm) 13.7 (s, 1H, COOH), 9.1 (d, J = 1.6 Hz, 1H, H_4_), 7.7 (q, J = 1.7 Hz, 2H, H_1_ and H_2_), 5.1 (s, 2H, H_5_), and 3.9 (s, 3H, H_3_) and the ^13^C NMR signals are displayed at δ (ppm) 168.7 (C_6_), 137.9 (C_4_), 124.1 (C_1_), 123.5 (C_2_), 59.9 (C_5_), 36.4 (C_3_).

**Figure 2:**
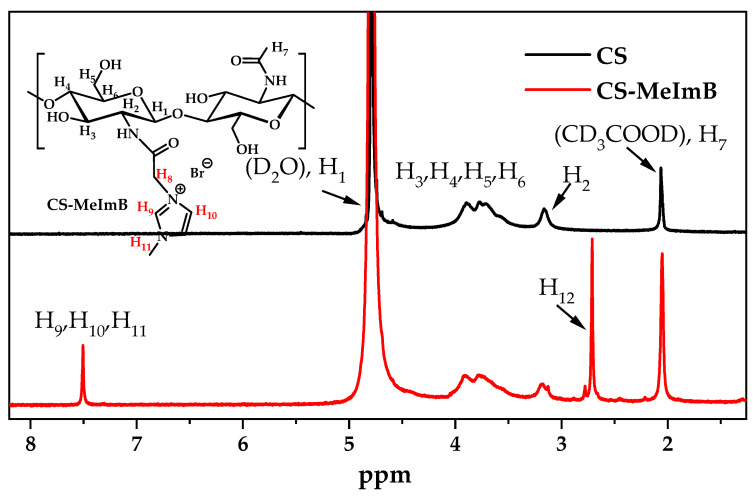
^1^H NMR spectra of CS and CS-MeImB.

After characterizing MeImB compound, the chemical modification of chitosan was performed. ^1^H NMR spectra of CS and its derivative were analyzed (see Figure 2). In the case of CS, a residual proton signal from the acetyl group is observed at 2.04 ppm overlapping with the methyl group of deuterated acetic acid, and a characteristic signal at 3.1 ppm corresponding to the H_2_ position of the polymer ring. The H_1_ signal overlaps with the D_2_O solvent peak, while signals from H_3_ to H_6_ appear in the range of 4.0 to 3.3 ppm [44].

In the case of CS-MeImB, the polysaccharide structure exhibits similar signals to those of CS and additional signals assigned to the imidazole ring, mainly at 7.45 ppm and at 2.58 ppm that corresponds to the three protons of CH_3_ [45,46]. These findings confirm the presence of new signals associated with the synthesized compound.

### 3.2. FTIR Analysis

**Figure 3:**
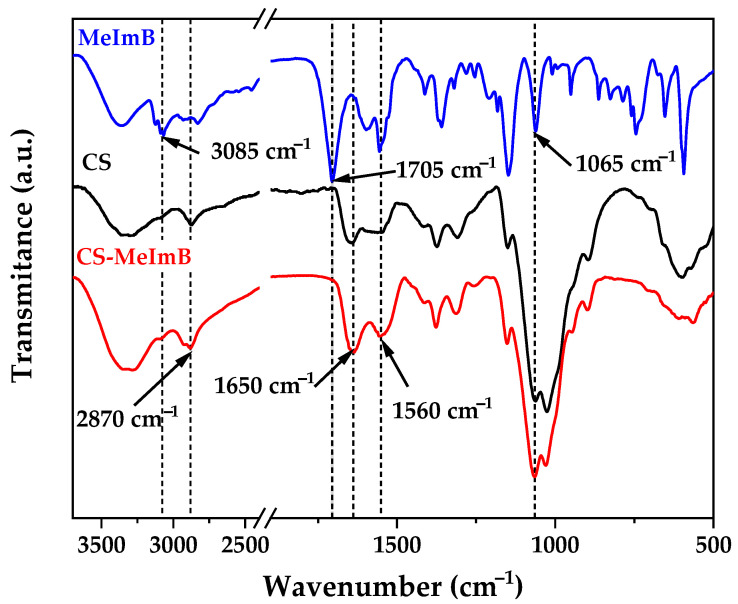
FTIR spectra of CS and CS-MeImB.

The chemical modification of CS with methyl imidazole was also studied by FTIR, the spectra of native CS, MeImB and the resulting CS-MeImB derivative are shown in Figure 3. A broad band at 3340 cm^−1^ corresponding to the –NH and –OH stretching vibrations and the intramolecular hydrogen bonds of the chitosan, followed by a band at 2870 cm^−1^ attributed to the stretching vibration of –CH groups [47] were observed. Two characteristic bands at 1650 cm^−1^ and 1560 cm^−1^ were seen, corresponding to the absorption of the –NH–CO– stretching vibration and the deformation vibration of NH_2_ groups, respectively [48].

On the other hand, for CS-MeImB, the characteristic bands of the CS were still observed, along with additional bands corresponding to the modification. A new band appeared at 3085 cm^−1^, corresponding to the stretching vibration of the –CH groups from the introduced methyl imidazole ring. Furthermore, the bands at 1650 cm^−1^ and 1560 cm^−1^, associated with the inserted amide groups and the deformation vibration of –NH–, both showing increased intensity and exhibiting a slight shift, due to the amino group of the imidazole ring [49].

### 3.3. Elemental Analysis

The degree of modification was calculated based on elemental analysis, and the results are presented in Table 2. The theoretical values of CS elemental analysis are also included for clarity. MeImB molecule presents in its elemental analysis 51.1% for C, 6.4% for H, and 19.9% for N; therefore, a C/N ratio of 2.6. Then, a significant decrease in the C/N ratio was observed after the modification of chitosan attributed to the insertion of the imidazole group. From these data the degree of substitution was found to be 57%. It is worth noting that the degree of acetylation (DA) remains constant, as the CS was not subjected to a prior deacetylation process.

### 3.4. X-Ray Diffraction (XRD)

**Figure 4:**
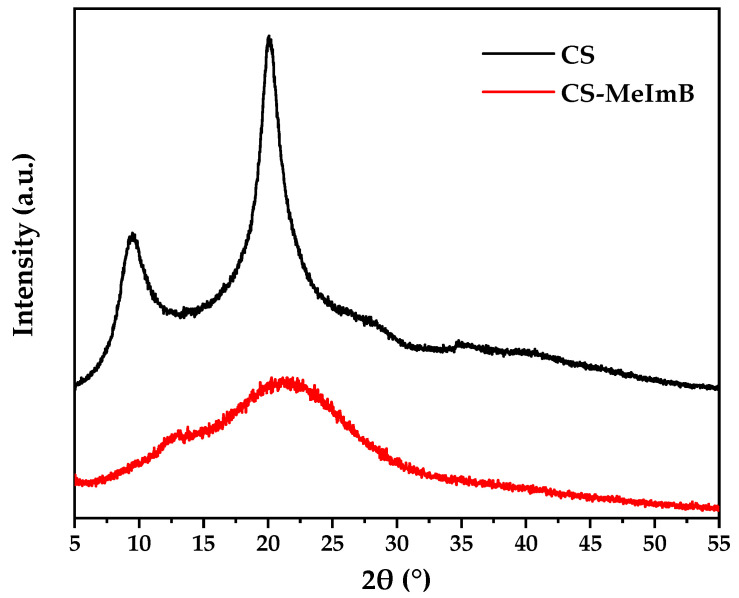
X-ray diffraction patterns of CS and CS-MeImB.

The X-ray diffraction patterns of CS and CS-MeImB are presented in Figure 4. For CS, two prominent peaks were observed at 9.4° and 20.0°, corresponding to the (020) and (110) planes of the crystalline structure, respectively [50].

After modification, the crystalline phase is destroyed; only a very small peak at 14° was observed besides the wider amorphous halo [51]. This could suggest that the imidazole ring retains some crystallinity within the modified structure. However, introducing polar groups disrupts the strong hydrogen bond between CS chains, inducing the disorganization of crystalline structure [52]. Consequently, the material exhibits a higher amorphous content and a reduced crystalline phase. The introduction of the polar molecule promotes strong covalent and non-covalent interactions among the hydroxyl functional groups of CS [53].

### 3.5. Thermogravimetric Analysis (TGA)

**Figure 5:**
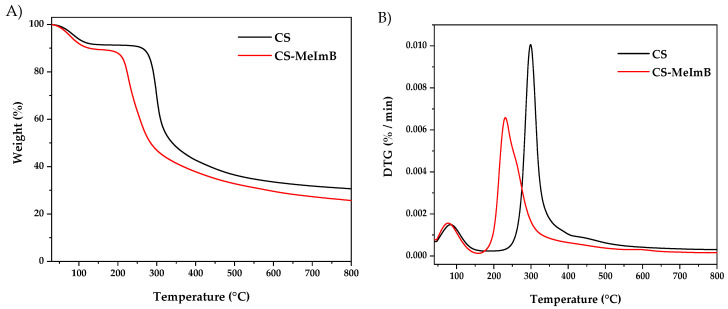
(**A**) Thermogravimetric curves of CS and CS-MeImB and (**B**) their derivatives (DTG).

The thermogravimetric analysis of CS and CS-MeImB revealed distinct thermal behavior for both samples (Figure 5). An initial weight loss of approximately 10% was observed in both cases, attributed to water loss, occurring between 40 °C and 150 °C, which is typical for highly hydrophilic polysaccharides [54].

Subsequently, in the case of the CS derivative, a second stage of thermal decomposition was evident, corresponding to the degradation of the organic matrix and the chemically attached methylimidazole groups, resulting in a 65% mass loss. This suggests that the introduced organic group as side chain with the formation of amide could be a starting site of breaking promotes the thermal degradation of CS, and decreasing the thermal stability of the final material [55,56]. For unmodified CS, the degradation stage of the glucosamine units occurred between 257 °C and 392 °C, leading to a total mass loss of approximately 60%. The carbonaceous residue is high due to the inert atmosphere used and is habitual found in polysaccharides and glycopolymers [57,58].

### 3.6. Differential Scanning Calorimetry (DSC)

**Figure 6:**
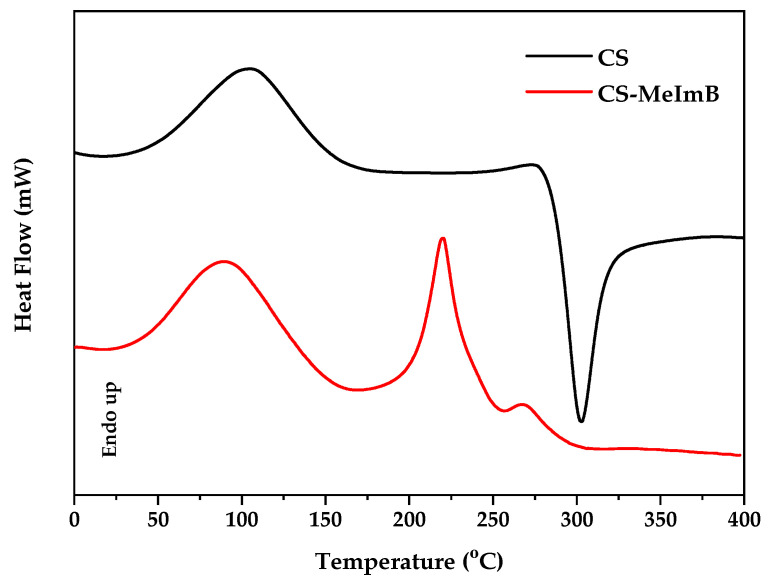
Differential scanning calorimetry curves of CS and CS-MeImB.

The thermal properties of CS and CS-MeImB were analyzed using DSC to understand the effects of the modification (Figure 6). Both samples exhibit a broad endothermic peak between 48 °C and approximately 150 °C, attributed to the evaporation of water bound to the polymer. In the case of CS, an exothermic peak also appears between 280 °C and 340 °C, corresponding to the decomposition of the main backbone of the polymer [59].

In the case of the CS-MeImB, the second peak appears around 214 °C as an endothermic process, corresponding to the decomposition of the complex formed between CS and the MeImB molecule [60,61]. Additionally, the formation of the derivative reduces the decomposition temperature of the CS backbone due to modifications in the natural organization of intermolecular bonds in CS-MeImB [62].

### 3.7. Antioxidant Activity of Chitosan Derivative

CS is known for its intrinsic antioxidant properties due to the presence of –OH– and –NH_2_ groups, which can donate hydrogen atoms to neutralize reactive species [49,63,64]. However, chemical modifications could alter these properties by affecting hydrogen bonding and electron delocalization.

Imidazole molecules are known for their antioxidant properties due to their ability to stabilize free radicals through electron donation and resonance effects [10,65]. Additionally, the inherent positive charge of imidazolium-functionalized compounds could enhance antioxidant activity by facilitating interactions with radical species [9,18]. To evaluate this possible enhancement, the antioxidant activity was quantified using the DPPH assay, with Trolox as the standard for calibration.

**Figure 7:**
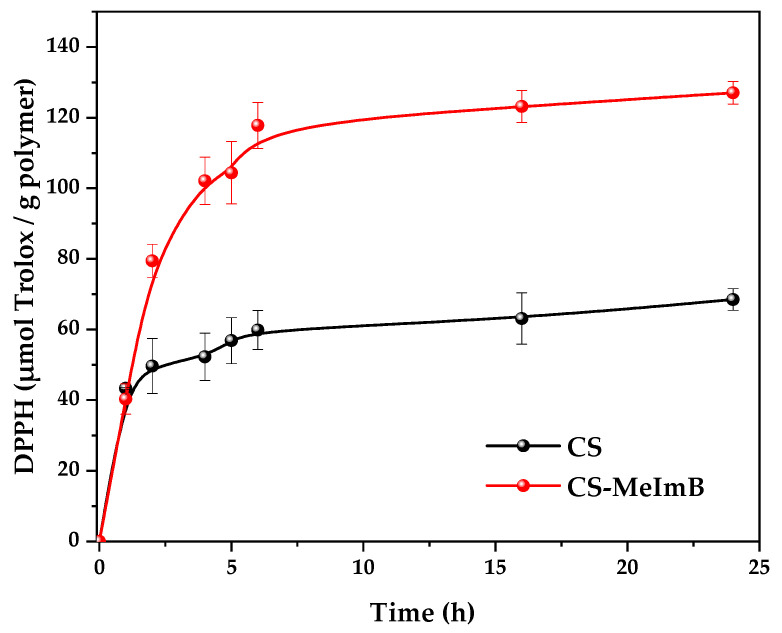
Antioxidant activity of CS and CS-MeImB versus time.

Figure 7 illustrates the DPPH radical scavenging capacity of CS and CS-MeImB. It is clearly appreciated that the antioxidant capacity is enhanced in the derivative with the functionalization of MeImB. For CS, the radical stabilization reaches its maximum after 5 h, achieving 58–60 μmol of Trolox per gram of polymer [66]. In contrast, CS-MeImB demonstrates faster DPPH scavenging kinetics, reaching a plateau within the same time but with double the capacity, reaching 120 μmol of Trolox per gram of polymer.

Heterocyclic groups have been extensively investigated to improve the antioxidant capacity of CS, with the imidazole group giving the most promising results, achieving nearly 90% inhibition at relatively low concentrations [67]. Thus, it can be concluded that the presence of the quaternary imidazole ring in CS-MeImB significantly improves the DPPH radical stabilization capacity, which explains its higher antioxidant activity and accelerated kinetics.

### 3.8. Antimicrobial Activity of CS-MeImB

**Figure 8:**
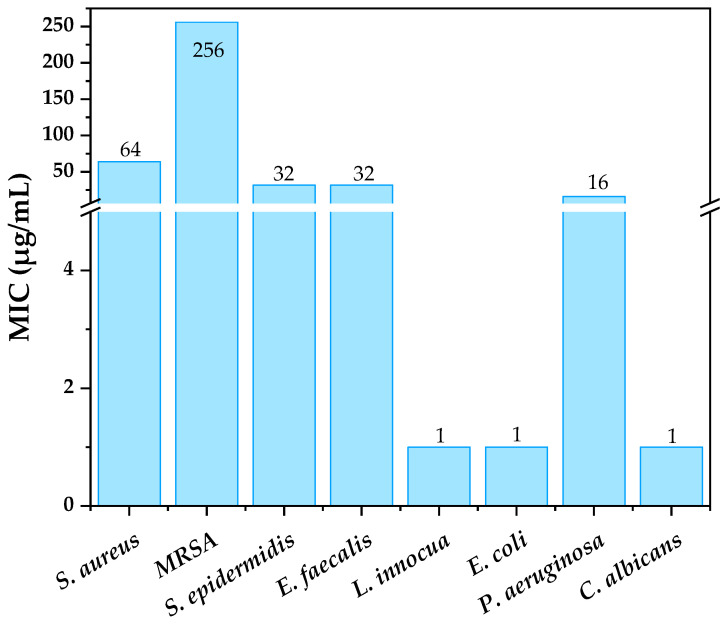
MIC values of CS-MeImB against different strains.

After proper characterization of the chemical structure and properties of CS-MeImB, its potential antimicrobial activity was also evaluated. In contrast to native CS, which is insoluble in aqueous media and forms aggregates at physiological pH, CS-MeImB demonstrates significant solubility under these conditions. This improved solubility appears from its cationic structure and net positive charge, which enable better dispersion at physiological pH and provide antimicrobial capabilities [18,62]. Indeed, the zeta potential, a measure of surface charge density, was determined to be 49 ± 5 mV. As mentioned above, this high positive charge density enhances not only its water stability but also its ability to interact with the negatively charged components of bacterial cell walls, potentially leading to greater antimicrobial efficacy. The combination of physiological solubility and a pronounced positive charge suggests that CS-MeImB might exhibit superior antimicrobial properties compared to CS. Certainly, this derivative presents a strong effectiveness against Gram-positive and negative bacteria, including resistant strains, and also against fungi, as can be seen in Figure 8, with MIC values remarkably low for the majority of the tested microorganisms. Similarly, other chitin derivatives containing imidazolium groups reported in the literature exhibited broad-spectrum antimicrobial activity [24,49].

### 3.9. Characterization of Films

Once the new compound has been characterized, the next step involves the preparation of CS films incorporating CS-MeImB. This derivative was added as an active component, aiming to enhance the properties of the resulting films. Therefore, the films underwent a wide physicochemical and biological characterization.

### 3.10. Thermogravimetric Analysis of Films

**Figure 9:**
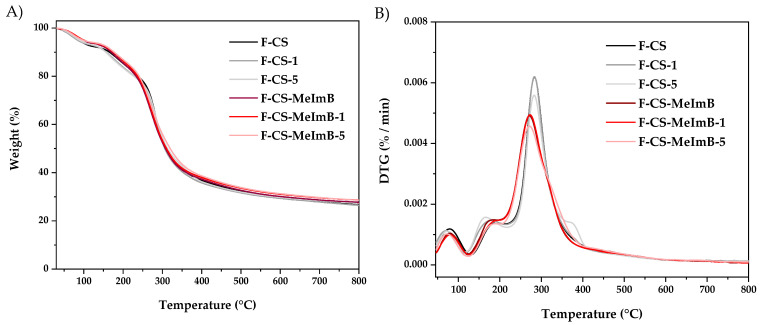
(**A**) Thermogravimetric curves of CS films and (**B**) its derivative (DTG).

The thermal decomposition of CS films containing CS-MeImB films, along with ChNws, was analyzed using TGA. The mass loss curves for all films and their derivative curves are shown in Figure 9A and Figure 9B, respectively. Within the studied temperature range, two main degradation steps can be observed, along with a shoulder in the second step [68].

In all cases, the first thermal event occurs from room temperature up to 120 °C, corresponding to the evaporation of absorbed water from the films due to their hydrophilic nature [69]. As the temperature increases, a shoulder appears around 190 °C, associated with glycerol evaporation, present in the formulations in a 15% [70]. This event is similar for all samples; however, in the case of CS-MeImB films, the shoulder is slightly shifted to a higher temperature. This shift could be attributed to the inherent positive charge of the MeImB moiety, which likely forms stronger hydrogen bonds with glycerol, requiring a higher temperature for its evaporation.

Finally, the second degradation step occurs between 220 °C and 350 °C, corresponding to the decomposition of CS main chain. Differences can be observed between the films made solely of CS and those containing 10% CS-MeImB, with the latter exhibiting lower thermal stability, as previously observed. Regarding the influence of ChNws incorporation, the addition of these nanocrystals did not seem to affect the thermal stability, presenting values of the maximum degradation temperature similar to that without ChNws. This fact was analogous in both CS and CS-MeImB films [71].

### 3.11. X-Ray Diffraction of the Films

**Figure 10:**
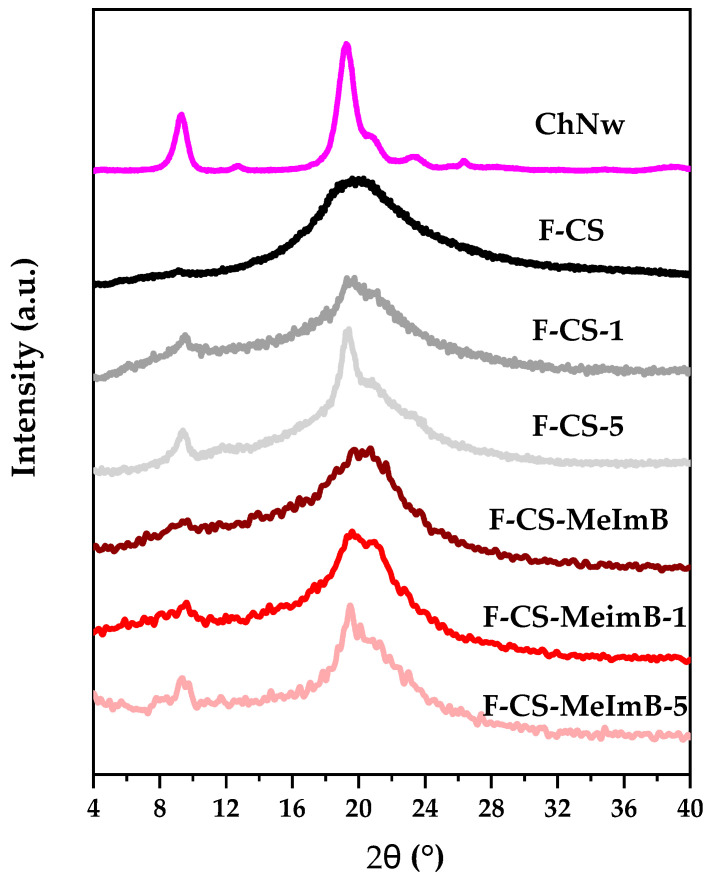
X-ray diffraction patterns of CS films.

X-ray diffraction analysis was performed to evaluate the crystalline structure of the films produced with CS and CS-MeImB. To further investigate the structural modifications induced by the chemical functionalization, the effect of incorporating the two different amounts of ChNws into the films was also studied. Films containing two different ChNw concentrations (1% and 5%) were analyzed to assess their influence on the crystallinity and potential interactions with the polymer matrix (Figure 10).

First, the crystalline structure of ChNw was characterized by its distinct peaks at 2θ = 8.9° (020 plane) and 19° (110 plane), along with a shoulder at 2θ = 20° (120 plane). This confirms that the internal crystalline structure of the nanowhiskers matches that of chitin powder [72].

Regarding the crystalline structure of the F-CS film, it appears predominantly amorphous, with a broad diffuse peak at 2θ = 19.7° and a barely perceptible peak at 2θ = 9.4° [73]. This suggests that dissolving CS in an acidic medium with Gly disrupts the formation of intramolecular hydrogen bonds, restricting the mobility of CS chains during film formation and consequently disturbing the crystalline regions of the polymer. When different amounts of ChNws were incorporated into pure CS films, the characteristic crystalline peaks of the nanocrystals at 2θ = 8.9° and 19° became more pronounced as their concentration increased, while the amorphous structure of the F-CS film with the plasticizer remained unchanged [74].

Incorporating CS-MeImB into the polymer matrix does not introduce notable structural changes. First, the peak at 2θ = 9.4° is maintained in both of the cases, with and without CS-MeImB. Also, the broad band at 2θ = 19.7° remains present, confirming the amorphous nature of the material [75]. Furthermore, as previously observed, the addition of ChNws enhances the intensity of its characteristic crystalline peaks, resulting in a more crystalline overall film [76].

### 3.12. Yellowness Index

The yellowing index (YI) was analyzed in the films due to its importance in preserving visual quality, ensuring product integrity against external factors, and meeting industry standards. UV-visible spectroscopy was employed to assess YI in the 380–780 nm range, using a 10 nm step width. Due to the inherent transparency of the films, transmittance measurements were used to evaluate changes in optical properties (see Table 3). The results indicate that the incorporation of ChNws led to an increase in film turbidity and a higher yellowing index, confirming that nanowhisker addition in a high proportion (5%) impacts the optical clarity of the films. However, when comparing CS and CS-MeImB films, no significant differences in YI were observed. This suggests that the chemical modification did not substantially alter the optical properties of the films.

From an industrial perspective, maintaining a low yellowing index is advantageous in packaging applications, as excessive yellowing may be undesirable to consumers. The results indicate that, despite the increased turbidity caused by ChNws, the overall visual appearance of the films remains within an acceptable range, making them suitable for potential packaging applications.

### 3.13. Water Vapor Transmission

The WVT of CS films and those incorporating CS-MeImB were evaluated with the different amounts of ChNws. In all cases, the WVT values were higher for CS films compared to films containing CS-MeImB (see Table 3). This difference could be attributed to the strong and active intermolecular interactions in the case of CS-MeImB derivative, which restrict the diffusion of water vapor through the polymeric matrix. The reduced WVT could also be related to the presence of the positively charged imidazole group, which promotes additional interactions, leading to a more compact and less permeable structure [77]. Furthermore, in both types of films, the WVT values do not vary with these ChNws content.

### 3.14. Mechanical Properties

The mechanical properties of the films were evaluated in terms of Young’s modulus, tensile strength, and elongation at break (see Table 3). The effect of incorporating 1% and 5% ChNws into both F-CS and F-CS-MeImB films was analyzed to determine their impact on film stiffness, strength, and flexibility.

For native CS based films, the addition of nanowhiskers led to an increase in Young’s modulus from 11.2 ± 1.6 MPa (F-CS) to 19.0 ± 2.1 MPa (F-CS-5), after the addition of ChNw, indicating enhanced stiffness due to the reinforcement effect. However, no significant changes were observed in tensile strength, remaining in the range of 24–29 MPa and suggesting that the intermolecular interactions between CS and ChNws did not considerably alter the film’s maximum resistance to deformation. Similarly, elongation at break remained unchanged, implying that the overall flexibility of the films was not significantly affected by the incorporation of nanowhiskers, as reported in the literature [78].

A similar trend was observed for the containing CS-MeImB films, where the incorporation of nanowhiskers at both 1% and 5% increased Young’s modulus, from 14.1 ± 0.8 MPa (F-CS-MeImB) to 19.5 ± 1.8 MPa (F-CS-MeImB-5); while tensile strength and elongation at break remained largely unaffected between 22 and 27 MPa. However, a comparison between CS and CS-MeImB films revealed some small differences. The Young’s modulus values were a bit higher for the F-CS-MeImB, suggesting that the chemical modification contributed to the increase in stiffness. In contrast, tensile strength values remained comparable between both film types, while elongation at break was considerably lower in CS-MeImB films compared to unmodified CS films.

The observed increase in stiffness with the incorporation of ChNws is aligned with previous studies, which have attributed this effect to hydrogen bonding interactions between polymer chains and the reinforcing nanowhiskers [79]. This reinforcement mechanism limits molecular mobility, resulting in stiffer films. Moreover, the lack of significant changes in tensile strength suggests that the interfacial adhesion between chitosan and chitin nanowhiskers is sufficient to maintain the film’s mechanical integrity without introducing substantial structural defects. The observed decrease in elongation at break in CS-MeImB films may be attributed to the combined effects of chemical modification and nanoparticles incorporation, which could lead to a more rigid polymer network with reduced flexibility.

### 3.15. Antioxidant Activity of Chitosan Films

**Figure 11:**
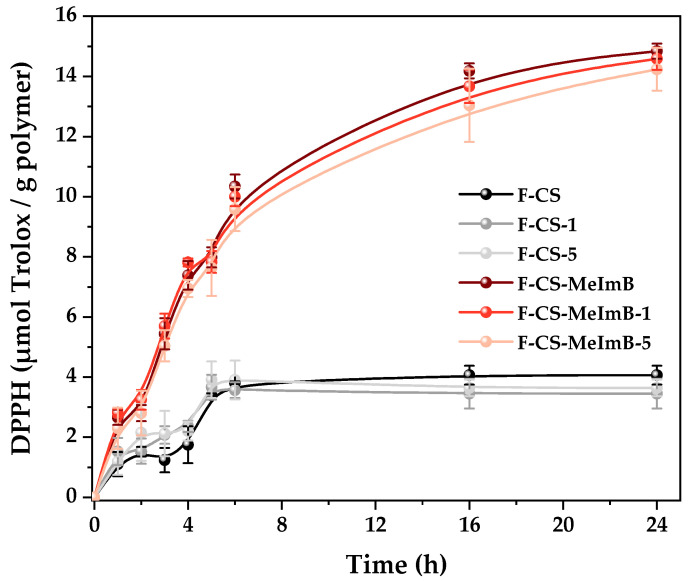
Antioxidant activity of CS films.

The antioxidant activity of the films was evaluated to determine the effect of CS-MeImB and the incorporation ChNws on their free radical scavenging capacity. The influence of CS-MeImB on the CS matrix is expected to affect the antioxidant behavior of the films by enhancing interactions with free radicals. To assess these effects, the antioxidant activity was measured using the DPPH radical scavenging assay, comparing the different film compositions (Figure 11).

The radical scavenging capacity of films containing only CS was relatively low, approximately 3 μmol Trolox per gram of film, reaching equilibrium after 5 h in all cases (F-CS, F-CS-1 and F-CS-5), regardless of the amount of ChNws added, as the values remain statistically similar.

In contrast, films made with CS-MeImB demonstrated a significantly higher capacity for scavenging DPPH radicals, reaching 14–15 μmol Trolox per gram of film, after approximately 20 h. This enhanced antioxidant activity was attributed to the increased positive charge and the electronic delocalization of the imidazole ring [80]. However, the incorporation of 1% or 5% nanowhiskers in these films slightly reduces their antioxidant activity, possibly due to steric effects limiting the accessibility to the active sites.

### 3.16. Antimicrobial Activity of Chitosan Films

**Figure 12:**
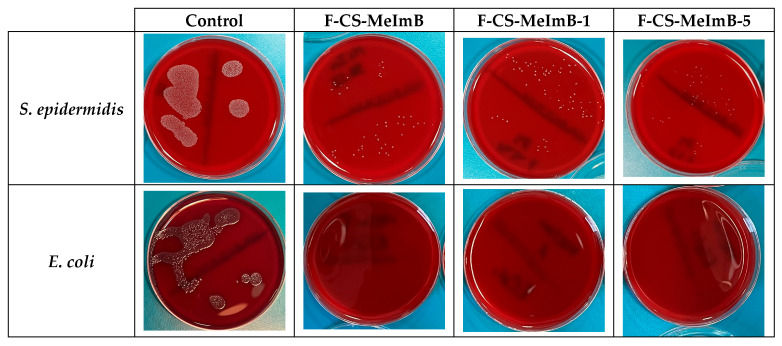
Images of the agar plates used for microbial colonies determination by plate counting method.

The evaluation of the antimicrobial activity of the films revealed clear differences between the unmodified CS films and those functionalized with MeImB (see Table 4). Films composed exclusively of CS exhibited insignificant antibacterial activity, except against *S. aureus*, where a moderate inhibitory effect was observed. However, no significant reduction in bacterial growth was detected for *P. aeruginosa*, *E. faecalis* or *E. coli.* In contrast, films containing active CS-MeImB demonstrated a great improvement in antimicrobial performance. This effect was particularly pronounced against Gram-negative bacteria, where complete bacterial elimination was observed in the case of *E. coli*. These findings highlight the effectiveness of MeImB-modified CS films, enhancing antimicrobial properties, particularly against Gram-negative bacterial strains. Examples of bacterial reduction in films are shown in Figure 12.

## 4. Conclusions

In this study, a water-soluble, strong antioxidant and antimicrobial imidazolium chitosan derivative, CS-MeImB, was obtained and incorporated as an active additive into CS-based films. The films were also reinforced with ChNws to improve mechanical and barrier properties. The functional CS films containing CS-MeImB demonstrated enhanced antioxidant properties and strong inhibitory effect against different strains, particularly against Gram-negative bacteria such as *E. coli* and *P. aeruginosa*. The incorporation of the CS-MeImB derivative slightly modified the mechanical properties, increasing Young’s modulus but decreases elongation at break. In addition, they exhibited notably lower water vapor transmission values compared to CS films, indicating an enhanced barrier effect resulting from the chemical modification. All these improvements were obtained without diminishing their optical properties. On the other hand, the incorporation of ChNw as fillers did not significantly modified their mechanical performance but facilitated their barrier properties.

All these characteristics make them promising materials for biodegradable packaging and other advanced applications where structural reinforcement and bioactivity are required.

## Figures and Tables

**Table 1:** Composition of chitosan-based films (%).

Sample	CS (%)	CS-MeImB (%)	ChNws (%)	Gly (%)
F-CS	85	-	0	15
F-CS-1	84	-	1	15
F-CS-5	80	-	5	15
F-CS-MeImB	75	10	0	15
F-CS-MeImB-1	74	10	1	15
F-CS-MeImB-5	70	10	5	15

**Table 2:** Percentage of carbon, hydrogen, and nitrogen, degree of acetylation, and degree of substitution of CS and CS-MeImB.

	C (%)	H (%)	N (%)	C/N	DA (%)	DS (%)
CS	40.6 ± 0.3	6.7 ± 0.3	7.2 ± 0.3	5.6	28	-
CS_theo_	44.3	6.9	8.0	5.5	25	
CS-MeImB	39.5 ± 0.3	6.5 ± 0.1	9.4 ± 0.1	4.2	28	57

**Table 3:** Yellow indexes (YI), water vapor transmission (WVT), mechanical properties of CS films elastic modulus (E), the maximum tensile strength (TS), and elongation at break (ε).

	YI (%)	WVT 10^−3^ (g/(d × m)	E (MPa)	TS (MPa)	ε (%)
F-CS	2.73 ± 0.16 ^a^	81.4 ± 5.4 ^a^	11.2 ± 1.6 ^a^	28.7 ± 3.5 ^a^	10.3 ± 3.0 ^a^
F-CS-1	3.16 ± 0.15 ^a^	78.6 ± 5.5 ^a^	13.9 ± 2.1 ^a^	24.0 ± 4.9 ^a^	6.4 ± 1.5 ^ab^
F-CS-5	6.34 ± 0.19 ^b^	70.4 ± 8.7 ^a^	19.0 ± 2.1 ^b^	24.3 ± 5.7 ^a^	8.9 ± 3.8 ^ab^
F-CS-MeImB	2.78 ± 0.15 ^a^	21.1 ± 0.9 ^b^	14.1 ± 0.8 ^a^	22.4 ± 5.3 ^a^	3.6 ± 2.1 ^c^
F-CS-MeImB-1	3.13 ± 0.16 ^a^	22.6 ± 1.5 ^b^	15.7 ± 1.0 ^ab^	28.0 ± 2.1 ^a^	2.8 ± 1.2 ^c^
F-CS-MeImB-5	5.09 ± 0.94 ^b^	23.5 ± 0.9 ^b^	19.5 ± 1.8 ^b^	26.8 ± 6.1 ^a^	1.8 ± 0.6 ^c^

Values with the same letter are not significantly different for Tukey test (Significance level of *p* ≤ 0.05).

**Table 4:** Antibacterial activity of the resulting CS based films estimated as bacterial reduction percentage.

	*S. aureus*	*S. epidermidis*	*P. aeruginosa*	*E. coli*
F-CS	85.3 ± 1.1 ^a^	-	-	-
F-CS-1	88.5 ± 1.4 ^b^	-	-	-
F-CS-5	79.7 ± 0.9 ^c^	-	-	-
F-CS-MeImB	85.5 ± 2.3 ^ab^	99.5 ± 2.1 ^a^	97.5 ± 1.7 ^a^	99.999 ± 0.001 ^a^
F-CS-MeImB-1	87.7 ± 2.7 ^ab^	99.8 ± 1.3 ^a^	99.1 ± 2.1 ^a^	99.999 ± 0.001 ^a^
F-CS-MeImB-5	92.1 ± 4.3 ^ab^	97.8 ± 2.6 ^a^	99.4 ± 1.5 ^a^	99.999 ± 0.001 ^a^

Values with the same letter are not significantly different for Tukey test (Significance level of *p* ≤ 0.05).

## Data Availability

The raw data supporting the conclusions of this article will be made available by the authors on request.
